# Efficient intra mode decision for low complexity HEVC screen content compression

**DOI:** 10.1371/journal.pone.0226900

**Published:** 2019-12-31

**Authors:** Qiuwen Zhang, Yongbo Zhao, Weiwei Zhang, Lijun Sun, Rijian Su

**Affiliations:** 1 College of Computer and Communication Engineering, Zhengzhou University of Light Industry, Zhengzhou, Henan, China; 2 School of Electrical Engineering, Henan University of Technology, Zhengzhou, Henan, China; Nanjing University of Information Science and Technology, CHINA

## Abstract

High efficiency video coding screen content coding (HEVC-SCC) extension is the latest HEVC development to improve the compression performance of screen content (SC) video. Similar to HEVC, the intra mode selection in HEVC-SCC is performed all the coding unit (CU) partitions to find the least rate distortion (RD) cost. Furthermore, additional intra tools are introduced to improve HEVC-SCC coding efficiency. However, these new tools could cause high computation complexity which restricts HEVC-SCC from ongoing applications. To solve the problem, an efficient intra mode decision for HEVC-SCC that adaptively utilizes the texture complexity of SC treeblock is proposed. The texture complexity of a SC treeblock is first analyzed according to the variation degree of the luminance value. And then, two efficient approaches are proposed based on the constructed model, which are early CU depth level determination and adaptive intra mode selection. Experimental results demonstrate that the proposed method can save 48.5% encoder runtime while keeping nearly the same coding efficiency as the HEVC-SCC encoders.

## 1. Introduction

With the development of wireless displays and mobile technologies, screen content (SC) video attracts more and more attentions in the last few years [1–4]. SC is different from the conventional camera-captured image and has mixed content consisting of graphics, text and natural pictures in the same image with higher resolution. For such different characteristics, SC video compression standard has been investigated by JCT-VC, and high efficiency video coding-screen content coding (HEVC-SCC) is formed as the extension of HEVC since March 2014 [5–8].

In the HEVC-SCC, additional tools (intra block copy (IBC) [9] and palette coding mode (PLT) [10], etc.) have been proposed to improve coding efficiency in SC video [11]. Here, IBC is a block matching method using fixed block size for better SC video compression, which is similar to the inter-prediction estimation. PLT is the sample in the Coding Unit (CU) which is represented by a small set (as the palette) of representative color values. Even though these newly added intra mode tools could achieve the good rate distortion (RD) performance, it will bring significant large computation complexity. Therefore, a fast intra mode decision algorithm without compromising RD efficiency needs to be developed for HEVC-SCC encoders.

A lot of fast methods [12–18] have been proposed to speed up the computation of natural video compression, e.g., HEVC achieves good runtime savings with negligible loss of RD performance. However, those methods basically consider the natural video captured by a real camera without exploring the unique signal properties of screen content coding. Screen content video typically contains less sharp edges, distinct colors, irregular motions and complex local textures. Conventional fast coding works are not applied to HEVC-SCC, where complexity reduction is intrinsically related to the newly intra structures for HEVC-SCC. It can be more efficient to save computational complexity by exploiting screen content characteristics.

Recently, several recent algorithms [19–32] have been reported to reduce the coding complexity of HEVC-SCC. A fast block copy search is investigated in [19] to speed up the HEVC-SCC intra coding process based on CU activity and regular mode cost. A fast intra partition method is presented in [20] based on coding bits to save coding time, in which some rules are used to decide the CU partition. An adaptive precision is exploited in [21] to reduce the computation load of the HEVC-SCC encoders by the hash block matching. A fast decision is designed in [22] to decrease residual coding complexity in transform process, in which intra block copy block with zero flag is enforced to be coded for skip mode. A fast intra coding approach which focuses on smooth regions is proposed in [23] to decrease the encoder complexity by skipping the IBC and PLT mode. A fast prediction is designed in [24] to decrease the number of intra modes from total 35 to 3. Fast intra and block matching is presented in [25] for HEVC-SCC encoders by exploiting block search size and temporal correlation. Fast CU and partition selection methods are employed in [26] and [27] based on machine learning to decrease the encoding time of the SCC compression. A fast intra prediction method is introduced in [28] to speed up the HEVC-SCC encoder by exploiting the characteristics of screen content, where CU in SCC is classified into screen CU and natural CU. A fast intra coding method is designed in [29] to reduce the complexity of HEVC-SCC based on statistical learning. A fast transcoding framework is presented in [30] to accelerate the SC coding based on statistical mode mapping. A fast CU decision is employed in [31] using the ensemble learning to speed up the SC compression. A fast intra mode selection is proposed in [32] to speed up encoding computation by using a sequential arrangement of mode classifiers. These methods were made to search a better trade-off between computational complexity and coding efficiency. However, most of the previous existing algorithms only optimize particular mode decision, while the information among mode prediction and partition characteristics in HEVC-SCC is not fully studied. The texture complexity of SC has not been explored in the CU partitioning and intra mode decision process. Although a few fast coding methods based on texture-based block classification are proposed to select proper prediction directions, there is still much room to further reduce the coding complexity of HEVC-SCC on the intra mode decision process.

To overcome this problem, this paper presents a fast intra mode decision method for HEVC-SCC encoders by utilizing the texture complexity of SC treeblock. The variation degree of luminance values in a treeblock is used to texture complexity properties of the current treeblock and adjust intra mode prediction strategies in HEVC-SCC encoders. All depth levels and intra modes are divided into three types, and different types of SC treeblock are used to assign different modes. The comparative experiment results show that the proposed method can significantly reduce the complexity with only 1.24% bitrate decrease for intra mode coding. The feature of the proposed algorithm is that unlike the previously existing methods based on mode correlation not only among CU partitioning, but also the intra mode selection of IBC and PLT. Further, the proposed algorithm reduces unnecessary CU depths and prediction modes based on a complexity parameter of current SC treeblock to derive the adaptive thresholds for efficient intra mode decision. As far as we know, there is almost no work similar to the proposed algorithm on the HEVC-SCC.

The rest of the paper is structured as follows. Section 2 provides the analysis on HEVC-SCC mode selection. Section 3 presents the proposed intra mode decision method in detail. Simulation results and conclusions are given in Section 4 and 5, respectively.

## 2. Observations and analysis

HEVC-SCC is developed for the compression of screen content sharing systems, which is an extension of the HEVC. Similar to HEVC, the mode decision procedure in HEVC-SCC tries all the prediction modes and depth levels to find the one in terms of least RD cost,

$$J_{mode}=\left(SSE_{luma}+\omega_{chroma}\cdot SSE_{chroma}\right)+\lambda_{mode}\cdot R_{mode}$$ (1)

Where *J*_*mode*_ is the RD cost function, *SSE*_*lima*_ and *SSE*_*chroma*_ are the distortion between the current treeblock and its reconstructed block of luma and chroma component, *ω*_*chroma*_ is the chroma parameter, *λ*_*mode*_ denotes the Lagrange multiplier, *R*_*mode*_ denotes bit rate cost used for HEVC-SCC encoders. This “try all and select the best” mode decision method achieves the good RD performance, but it leads to significant high complexity. Furthermore, additional tools including IBC and PLT have been developed for HEVC-SCC to improve coding efficiency. Compared with conventional HEVC mode prediction, all the IBC and PLT modes are added to the RD search list in HEVC-SCC encoders. These techniques result in extremely huge complexity, which obstructs HEVC-SCC encoders from real-time application. In the current test model, test IBC and PLT modes together with traditional HEVC intra prediction are a big consuming part of HEVC-SCC encoders due to high computational complexity for full RD cost calculation.

In fact, larger CUs using traditional HEVC intra mode are suitable for treeblock in flat or homogeneous region, while IBC and PLT modes are more likely to be chosen for the treeblock with sharper object edges or discontinuous-tone screen content region [28]. It is shown that the mode prediction of HEVC-SCC should be adaptively determined, which based on the SC texture content characteristic of current CU. Therefore, if we can exploit texture content characteristic to determine some modes which is rarely used in HEVC-SCC intra prediction, the full RD costs calculation can be effectively skipped in the whole coding process, and the complexity of HEVC-SCC could be significantly reduced.

We encode 14 SC test sequences jointly recommended by the experts from JCT_VC with different spatial resolutions of 2560×1440 (“Basketball_Screen” and “MissionControlClip2”), 1920×1080(such as “sc_flyingGraphics” and “sc_desktop” etc.), and 1280×720 (such as “sc_web_browsing” and “sc_map” etc.). Among these sequences, four categories of sequences are used in the experiments, namely “text and graphics with motion (TGM)”, “mixed content (M)”, “animation (A)”, and “camera-captured content (CC)” category. Test videos are available from this location: ftp://hevc@ftp.tnt.uni-hannover.de/testsequences. Experimental conditions are listed as follows: Loss coding, QP is chosen with 20, 25, 30, and 35, the number of coding frames is 50, all-intra configurations.

Table 1 gives the optimal intra mode distribution of the HEVC-SCC encoders. It is observed that the probabilities of choosing IBC, PLT, Planar, DC, Horizontal, and Vertical Mode are 22.8%, 5.8%, 17.4%, 10.1%, 14.4% and 19.9%, respectively, and the probability of other intra modes (Angular 2–9, 11–25, and 27–34) is equal to 9.7%. Note that, the percentage of choosing Horizontal and Vertical Mode cover are 14.4% and 19.9%, respectively. Especially for low bitrate, more than 50% of SCC treeblocks are coded with these two modes (QP = 35). This is reasonable since screen content video is dominated by purely horizontal and vertical patterns. It is noticed from experimental results in Table 1 that IBC mode and PLT mode utilization decrease as QP increase. The reason is that large bitrate (such QP = 20) has a higher likelihood to select good matches. However, the IBC and PLT mode occur very frequently for large homogeneous and smoothly-varying regions [28]. On the other hand, the IBC and PLT mode are rarely chosen for CUs in screen content blocks that generated by computers. If the optimal intra modes can be decided at the early stage, the wasteful search process of IBC mode, PLT mode and HEVC intra mode will be skipped, and thus a huge amount of complexity can be significantly reduced in HEVC-SCC.

**Table 1:** Intra mode distribution of the HEVC-SCC.

QP	IBC Mode(%)	PLT Mode(%)	Planar Mode(%)	DC Mode(%)	Horizontal Mode(%)	Vertical Mode(%)	Other intra Modes(%)
20	29.1	8.3	15.9	9.7	11.4	10.8	14.8
25	25.7	6.9	14.7	10.1	15.1	13.8	13.7
30	20.9	4.6	16.3	14.9	18.7	17.2	7.4
35	15.5	3.2	22.8	5.5	12.4	37.7	2.9
Average	22.8	5.8	17.4	10.1	14.4	19.9	9.7

Other intra modes are Angular modes 2–9, 11–25, and 27–34.

With the similar spatial characteristic, the texture complexity of a SC treeblock is strongly related to that of its luminance value. To characterize this correlation, the variation degree of luminance value in a SC treeblock is defined as follows,

$$LV\left(x,y\right)=\frac{1}{W\times H}\sum_{x=1}^{W}\sum_{y=1}^{H}{\left(L_{r}\left(x,y\right)-L_{a}\left(x,y\right)\right)}^{2}$$ (2)

where *LV* represents the different luminance parameter of current SC treeblock, *L*_*r*_(*x*, *y*) represents current treeblock luminance value, and *L*_*a*_(*x*, *y*) represents the average luminance value of current CU. If the different luminance values of current CU change drastically, a large value of *LV* is obtained.

Generally, the more complexity of SC treeblock has, the larger value of *LV* will be. According to *LV*, each SC treeblock is divided into homogenous, middle or complex texture region as:

$$\{\begin{array}{rr} LV\leq S_{h} & SCtreeblock\in homogenousregion\\ S_{s}<LV<S_{c} & SCtreeblock\in middletextureregion\\ LV\geq S_{c} & SCtreeblock\in complextextureregion \end{array}$$ (3)

where *S*_*h*_ and *S*_*c*_ are texture-weight factors. The selection of the threshold *S*_*h*_ and *S*_*c*_ should efficiently reduce the HEVC-SSC computational complexity, while they can keep the same RD performance and high accuracy in intra mode decision. Based on extensive experiments, the thresholds *S*_*h*_ and *S*_*c*_ are set to 30 and 60, respectively, which achieve a good and consistent performance on a variety of test SC sequences with different characteristics. In this paper, the coding information from luminance value variation is extracted to analyze intra prediction mode characteristics of the current treeblock and adjust the HEVC-SCC mode decision process.

## 3. Proposed intra mode decision method

### 3.1 Early CU depth determination

Similar to HEVC, the ME process in HEVC-SCC encoders is performed using all depth levels to select the best mode, and HEVC-SCC makes the “try all and select the best” method for each SC treeblock. However, this technique leads to an extremely high complexity. Thus, the exhaustive SCC which is fixed depth level range compression is inefficient, and fast CU depth determination is very desirable for HEVC-SCC encoders.

In HEVC-SCC, the maximum treeblock size is set to 64, and the CU depth range is 0 to 3. The CU depth level has a fixed range for a whole encoding process. In fact, small depth level is chosen for treeblocks with static region, while large depth level is suitable for treeblocks with complex motion region. It is observed that the depth level value of “0” occurs very frequently for the CUs in the static region. On the other hand, the depth level value of “0” is rarely chosen for CUs with complex motion [33]. These results show that the depth range in HEVC-SCC can be adaptively determined by complexity characteristics of SC treeblocks.

According to Eq (3), each SC treeblock can be classified into three types, which are homogenous treeblock, middle texture treeblock and complex texture treeblock. By exploiting the above test conditions and test sequences in Section 2, the depth distribution of HEVC-SCC could be analyzed for three types of SC treeblocks.

Table 2 gives the depth level distribution of HEVC-SCC for three types of treeblocks, in which “Depth 0”, “Depth 1”, “Depth 2” and “Depth 3” are the depth level values of SC treeblocks. It can be observed that for treeblocks in homogenous region, about 61.2% of SC treeblocks select the optimal depth level with “0” (CU size 64×64), and about 34.0% of SC treeblocks select the optimal depth level with “1” (CU size 32×32). Thus, if the maximum value of depth is set to “1”, it will cover about 95.2% of SC treeblocks. The intra mode prediction on depth value of “2” and “3” can be skipped. For treeblocks with middle texture region, about 94.6% of SC treeblocks select depth levels with “0”, “1” and “2” (CU size 64×64, 32×32, and 16×16). If the value of depth level is in the range of “0” to “2”, it will cover about 94.6% of SC treeblocks. For treeblocks with complex texture region, the probability of choosing the depth values of “2” and “3” (16×16 and 8×8) in HEVC-SCC is more than 91.6%, and thus depth levels of both “0” and “1” can be skipped. Based on that analysis, the optimal depth levels that will be tested in HEVC-SCC for three types SC treeblocks are given in Table 3. With the proposed early depth level range determination algorithm, most of SC treeblocks in HEVC-SCC intra prediction can skip one or two depth values.

**Table 2:** Depth distribution for three types of SC treeblocks.

Sequences	Homogeneous region				Middle texture region				Complex texture region			
	Depth 0 (%)	Depth 1 (%)	Depth 2 (%)	Depth 3 (%)	Depth 0 (%)	Depth 1 (%)	Depth 2 (%)	Depth 3 (%)	Depth 0 (%)	Depth 1 (%)	Depth 2 (%)	Depth 3 (%)
sc_flyingGraphics	68.1	28.9	2.2	0.8	22.1	38.2	35.1	4.6	1.4	10.2	39.3	48.1
sc_desktop	61.4	34.1	3.1	1.4	17.7	40.7	36.7	4.9	0.9	7.6	37.9	53.6
sc_console	58.7	36.2	3.3	1.8	15.9	40.9	36.5	6.7	1.1	6.1	38.6	54.2
ChineseEditing	62.2	34.7	2.4	0.7	18.6	40.4	35.6	5.4	1.3	5.8	40.2	52.7
MissionControlClip3	56.1	38.4	3.9	1.6	13.1	42.7	37.9	6.3	0.5	4.6	34.2	60.7
EBURainFruits	65.8	31.6	2.1	0.5	21.8	40.9	34.2	3.1	1.6	9.1	33.7	55.6
Kimono1	69.8	27.6	1.9	0.7	24.8	37.9	38.2	2.9	2.4	11.2	42.7	43.7
sc_web_browsing	63.7	32.2	2.9	1.2	28.4	41.2	24.1	4.2	1.2	6.9	38.7	53.2
sc_map	58.6	35.2	4.1	2.1	14.1	38.9	40.1	6.9	0.9	5.2	37.5	56.4
sc_programming	63.1	32.2	3.4	1.3	18.1	43.8	34.2	3.9	1.3	6.4	40.2	52.1
sc_SlideShow	54.3	38.3	5.1	2.3	12.4	36.6	43.2	7.8	0.7	4.9	33.7	60.7
sc_robot	59.2	34.6	4.3	1.9	15.2	37.7	41.3	5.8	1.3	6.9	41.2	50.6
Basketball_Screen	56.4	36.9	4.5	2.2	13.2	39.2	40.9	6.7	1.6	6.7	39.9	51.8
MissionControlClip2	60.3	34.1	3.8	1.8	15.8	38.9	39.4	5.9	1.9	7.2	41.6	49.3
Average	61.2	34.0	3.4	1.5	17.9	39.9	37.0	5.4	1.3	7.1	38.5	53.1

**Table 3:** Candidate depth levels of HEVC-SCC encoders for three types treeblocks.

Screen content treeblock	Candidate depth levels	[*Depth*_min_, *Depth*_Max_]
Homogenous region	0,1	[0, 1]
Middle texture region	0,1,2	[0, 2]
Complex texture region	2,3	[2, 3]

### 3.2 Adaptive intra mode selection

In the HEVC-SCC encoders, two newly intra modes, e.g., IBC and PLT, are applied for SC coding. Compared with HEVC, after the selection of the best one in the HEVC mode prediction, all IBC and PLT mode are added to HEVC-SCC encoder. This method will cost extremely high computational complexity.

**Fig 1:**
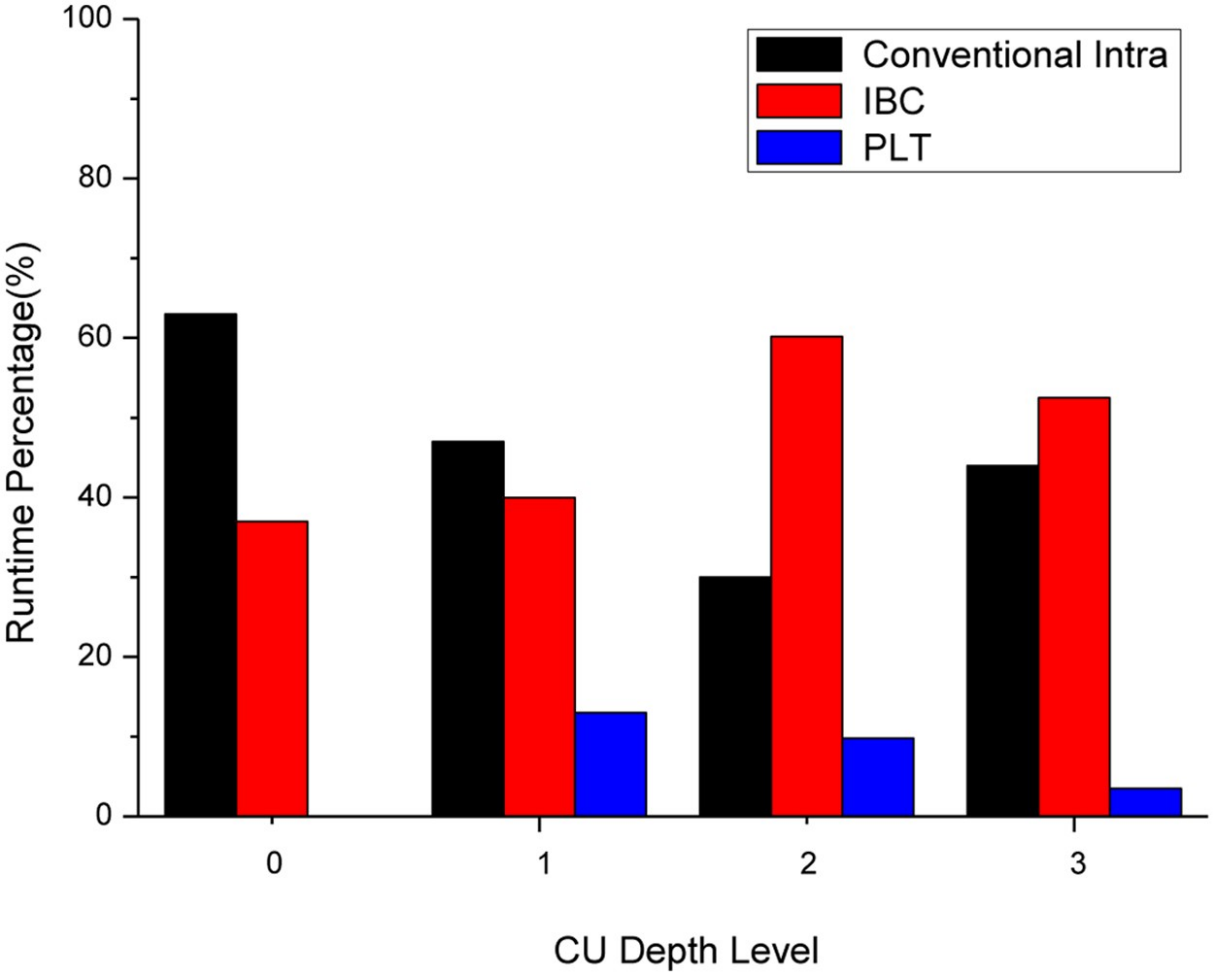
Runtime division in HEVC-SCC intra coding.

Fig 1 shows the runtime analysis of HEVC-SCC intra mode prediction. It is observed from Fig 1 that the major computation complexity is consumed by conventional HEVC intra modes and IBC. The PLT is a smaller percentage on all the depth levels. The PLT mode computation complexity at depth level 0 is vain since PLT mode is disabled at CU size 64×64. It also can be observed from the figure that the IBC utilization increases with the increase of depth value. This is because that the larger CU depth values have higher likelihood to search precise matches. Moreover, the new developed tools (IBC and PLT modes) represent 56% coding time of the HEVC-SCC intra mode coding, while the conventional HEVC has the remaining 44%. Experimental results analysis shows that testing the IBC and PLT mode become the most computationally intensive part of HEVC-SCC encoders. Therefore, the complexity of the IBC and PLT mode should be reduced significantly while the same coding performance is nearly maintained.

In the current HEVC-SCC, two newly developed modes: IBC and PLT mode are mainly for the effective compressing of SC video, while they might be inefficient for the homogenous texture region, and the HEVC intra modes (Planar and DC mode) can work well. IBC mode copies the treeblocks from the reconstructed area in the same frame, which is an inter-alike block matching coding tools. Repeated pattern is copied from the area with block vectors. It is mainly effective for the regions with complex texture and sharp edges. Similarly, for PLT mode, two tables are created to store major colors on the palette predictor. It is effective for the blocks with major colors. Based on the aforementioned analysis, it is noticed that most of the intra modes in HEVC-SCC should be skipped when the current treeblock is homogenous. In traditional HEVC intra coding, Planar and DC mode are often selected as the best mode for treeblocks with slowly varying values. It can be seen that when the best mode is Planar or DC mode, the treeblock is very likely to be the simple or homogeneous area. Meanwhile, both IBC and PLT mode are designed for SC video transition, which are inefficient for homogeneous region. Thus, most of IBC and PLT mode search can be skipped in homogeneous region. Based on these observations, if a treeblock contains homogeneous region, we only choose Planar and DC for candidate intra mode in HEVC-SCC coding.

To evaluate the performance of proposed selection method for treeblock with homogenous region, extensive experiments have been performed. Table 4 gives the accuracy of the skipping Horizontal mode, Vertical mode, and Angular modes 2–9, 11–25, 27–34, IBC and PLT mode for treeblock with homogenous region. The accuracy of the proposed selection method is more than 98.3% (from 98.3% to 99.7%). Although the proposed method misses some cases when skipping IBC and PLT mode is still chosen as the best mode in HEVC-SCC, the loss rate which is less than 1.7% is negligible.

**Table 4:** Accuracy of the proposed selection method for treeblocks with homogenous region.

Sequences	QP = 20 (%)	QP = 25 (%)	QP = 30 (%)	QP = 35 (%)
sc_flyingGraphics	98.5	98.9	99.2	99.5
sc_desktop	97.8	98.2	98.8	99.2
sc_console	98.2	98.7	99.1	99.3
ChineseEditing	97.6	98.4	98.7	99.1
MissionControlClip3	98.9	99.1	99.3	99.5
EBURainFruits	99.2	99.5	99.7	99.7
Kimono1	97.8	98.4	98.7	98.9
sc_web_browsing	98.1	98.5	98.8	99.0
sc_map	97.6	98.2	98.5	98.7
sc_programming	97.9	98.4	98.7	98.8
sc_SlideShow	98.2	98.6	98.9	99.2
sc_robot	98.3	98.7	98.9	99.1
Basketball_Screen	98.9	99.2	99.4	99.5
MissionControlClip2	98.6	99.1	99.3	99.4
Average	98.3	98.7	99.0	99.2

Tables 5 and 6 show the intra mode distribution for treeblocks in middle texture and complex texture region. It can be observed from Table 5 that for a treeblock in the middle texture region, the probabilities of choosing IBC mode, Planar mode, DC mode, Horizontal mode, and Vertical mode are 13.9%, 26.3%, 14.0%, 18.2% and 20.4%, respectively. The probability of PLT mode and other intra modes is less than 4.9%. For the sequence of “EBURainFruits” and “Kimono1”, the percentage of choosing IBC mode is very low, 2.5%, because it’s the camera-captured content, and the IBC mode is developed for computer generated contents coding. The percentage of optimal intra modes (IBC, Planar, DC, Horizontal, and Vertical mode) is covered 92.6% candidate modes. Thus, from simulation results of Table 5 we can conclude that a treeblock in the middle texture region is likely to choose IBC, Planar, DC, Horizontal, and Vertical mode in HEVC-SCC encoders. For a treeblock of complex texture region, the probabilities of choosing Planar and DC mode are no more than 2.8% in Table 6. The total probabilities of IBC, PLT, Horizontal, Vertical and other intra modes are 95.8%. Thus, it is not necessary to run Planar and DC in the complex texture region. Based on the above analysis, the adaptive intra mode selection that will be tested in HEVC-SCC encoders is summarized in Table 7.

**Table 5:** Intra mode distribution for treeblock with middle texture region.

Sequences	IBC Mode(%)	PLT Mode(%)	Planar Mode(%)	DC Mode(%)	Horizontal Mode(%)	Vertical Mode(%)	Other intra Modes(%)
sc_flyingGraphics	17.3	3.1	22.1	13.2	18.2	21.2	4.9
sc_desktop	16.8	2.7	24.6	13.5	17.7	20.3	4.4
sc_console	17.9	3.4	20.8	14.1	18.2	21.5	4.1
ChineseEditing	15.2	2.3	23.4	12.8	21.7	22.1	2.5
MissionControlClip3	13.8	2.5	27.1	12.1	18.3	20.8	5.4
EBURainFruits	1.5	0.4	36.9	18.4	16.3	18.9	7.6
Kimono1	1.2	0.2	38.2	15.8	17.2	19.2	8.2
sc_web_browsing	18.2	3.5	22.3	13.5	18.5	21.1	2.9
sc_map	16.4	2.9	24.7	12.4	18.3	20.6	4.7
sc_programming	14.9	2.7	26.7	14.8	17.2	19.3	4.4
sc_SlideShow	15.7	3.2	24.8	10.9	19.7	22.3	3.4
sc_robot	18.7	3.6	22.6	12.7	17.9	19.3	5.2
Basketball_Screen	12.4	1.9	26.8	15.2	18.3	19.8	5.6
MissionControlClip2	13.8	2.3	25.8	16.2	17.6	18.9	5.4
Average	13.9	2.5	26.3	14.0	18.2	20.4	4.9

**Table 6:** Intra mode distribution for treeblock with complex texture region.

Sequences	IBC Mode(%)	PLT Mode(%)	Planar Mode(%)	DC Mode(%)	Horizontal Mode(%)	Vertical Mode(%)	Other intra Modes(%)
sc_flyingGraphics	34.9	8.9	2.1	1.3	15.1	18.2	19.5
sc_desktop	33.6	7.8	2.7	1.5	14.5	17.8	22.1
sc_console	36.5	9.6	1.8	0.7	16.2	19.1	16.1
ChineseEditing	31.3	7.4	2.9	1.4	18.6	21.2	17.2
MissionControlClip3	28.7	6.3	3.2	1.8	15.8	17.6	26.6
EBURainFruits	2.5	0.9	4.9	2.3	16.7	18.2	54.5
Kimono1	2.2	0.6	5.2	2.5	15.6	17.3	56.6
sc_web_browsing	37.3	9.3	1.8	0.9	16.9	19.5	14.3
sc_map	35.6	8.5	2.2	1.2	17.1	19.8	15.6
sc_programming	32.5	8.1	2.7	1.4	18.3	20.5	16.5
sc_SlideShow	34.2	7.7	2.3	1.1	15.3	17.2	22.2
sc_robot	38.6	9.7	1.4	0.6	16.6	18.5	14.6
Basketball_Screen	30.1	6.2	3.4	1.9	18.2	20.3	19.9
MissionControlClip2	31.2	7.1	3.2	1.5	19.1	21.2	16.7
Average	29.2	7.0	2.8	1.4	16.7	19.0	23.7

**Table 7:** Adaptive intra modes of HEVC-SCC encoders for three types treeblock.

Screen content treeblock type	Candidate intra mode
Homogenous region	Planar, and DC mode
Middle texture region	IBC mode, Planar, DC, Horizontal, and Vertical mode
Complex texture region	IBC, PLT, Horizontal, Vertical and other intra modes

### 3.3. Overall method

According to the above analysis, the proposed method is to adjust various steps of intra mode decision based on the texture complexity of SC treeblocks. The proposed overall method including early CU depth level determination and adaptive intra mode selection is shown as follows:

Step 1) Start intra mode prediction.
Step 2) Compute *LV*(*i*, *j*) based on (2), *S*_*h*_ and *S*_*c*_ for classification using (3), classify the SC into the homogenous, middle texture and complex texture region.
Step 3) Test early CU depth level determination. If current SC belongs to homogenous region, the optimal depth range is “0” to“1”; if current SC belongs to middle texture region, the optimal depth range is “0” to“2”; else if SC belongs to complex texture region, the optimal depth range is set to be “2” to“3”.
Step 4) Adaptive intra mode selection. If the SC treeblock belongs to homogenous region, Planar and DC mode are only used for intra coding; when the SC treeblock belongs to middle texture region, the optimal modes are IBC, Planar, DC, Horizontal, and Vertical mode. If the SC treeblock belongs to complex texture region, the optimal modes are IBC, PLT, Horizontal, Vertical and other intra modes.
Step 5) Determine the best intra mode.

## 4. Experimental results

To evaluate the performance of the proposed method, the experiments are implemented on the recent HEVC-SCC reference encoder (SCM 5.2) [34]. The proposed method is tested on fourteen sequences with different resolutions and motion activities in Table 8. Test videos are available from this location: ftp://hevc@ftp.tnt.uni-hannover.de/testsequences. All the simulations are performed by the SCC CTC [35]. Test conditions are listed as follows: Loss coding, all-intra configurations, four QPs are chosen with 22, 27, 32, and 37, the number of test coding frames is 120, and only YUV444 format results are reported by the computer space limit. In this section, we compare the proposed low complexity method in Tables 9–11 with the original HEVC-SCC and the state-of-the-art methods [25, 27, 28], where the compression efficiency is measured by BDPSNR and BDBR [36]. The “Dtime (%)” is used to represent the runtime saving in percentage,

$$\text{Dtime}=\frac{Time_{proposed}-Time_{origional}}{Time_{origional}}\times 100\%$$ (4)

where *Time*_*proposed*_ and *Time*_*origional*_ denote the encoding runtime of proposed scheme and original method for same test sequence, respectively.

**Table 8:** Test sequences information in HEVC-SCC CTC.

Sequences	Resolution	Category	Fps	Frames to be encoded
sc_flyingGraphics	1920×1080	TGM	60	0–119
sc_desktop	1920×1080	TGM	60	0–119
sc_console	1920×1080	TGM	60	0–119
ChineseEditing	1920×1080	TGM	60	0–119
MissionControlClip3	1920×1080	M	60	0–119
EBURainFruits	1920×1080	CC	50	0–119
Kimono1	1920×1080	CC	24	0–119
sc_web_browsing	1280×720	TGM	30	0–119
sc_map	1280×720	TGM	60	0–119
sc_programming	1280×720	TGM	60	0–119
sc_SlideShow	1280×720	TGM	20	0–119
sc_robot	1280×720	A	30	0–119
Basketball_Screen	2560×1440	M	60	322–441
MissionControlClip2	2560×1440	M	60	120–239

TGM: Text and graphics with motion; M: mixed content; CC: camera-captured content; A: animation

**Table 9:** Results of the proposed individual method.

Sequences	ECUDLD			ADIMD		
	BDBR (%)	BDPSNR (dB)	Dtime (%)	BDBR (%)	BDPSNR (dB)	Dtime (%)
sc_flyingGraphics	1.48	-0.18	-34.4	0.34	-0.03	-21.4
sc_desktop	1.12	-0.12	-38.1	0.21	-0.02	-24.1
sc_console	0.64	-0.08	-35.9	0.17	-0.02	-22.5
ChineseEditing	2.42	-0.24	-31.6	0.52	-0.06	-19.6
MissionControlClip3	0.58	-0.08	-32.4	0.09	-0.01	-20.4
EBURainFruits	0.22	-0.02	-29.8	0.12	-0.01	-18.9
Kimono1	0.28	-0.02	-46.7	0.00	-0.00	-29.1
sc_web_browsing	3.56	-0.38	-40.8	1.15	-0.12	-27.6
sc_map	0.84	-0.10	-39.1	0.29	-0.03	-26.9
sc_programming	0.62	-0.08	-37.5	0.19	-0.02	-25.3
sc_SlideShow	2.12	-0.22	-54.2	0.81	-0.09	-31.7
sc_robot	0.12	-0.02	-25.9	0.08	-0.01	-17.8
Basketball_Screen	0.51	-0.06	-38.9	0.41	-0.04	-24.9
MissionControlClip2	0.68	-0.08	-40.6	0.48	-0.05	-25.4
Average	1.08	-0.12	-37.6	0.35	-0.04	-24.0

**Table 10:** Results of the proposed overall method.

Sequences	BDBR (%)	BDPSNR (dB)	Dtime (%)
sc_flyingGraphics	1.68	-0.19	-43.9
sc_desktop	1.22	-0.13	-48.7
sc_console	0.71	-0.09	-46.5
ChineseEditing	2.69	-0.26	-41.4
MissionControlClip3	0.61	-0.08	-42.5
EBURainFruits	0.25	-0.02	-39.8
Kimono1	0.29	-0.02	-59.3
sc_web_browsing	4.12	-0.44	-53.1
sc_map	0.93	-0.11	-51.6
sc_programming	0.85	-0.09	-48.7
sc_SlideShow	2.31	-0.25	-66.3
sc_robot	0.16	-0.02	-35.2
Basketball_Screen	0.63	-0.08	-49.6
MissionControlClip2	0.89	-0.11	-51.7
Average	1.24	-0.14	-48.5

**Table 11:** Coding performance evaluations for the proposed algorithm in four categories of test sequences.

Category	ECUDLD			ADIMD			Overall algorithm		
	BDBR (%)	BDPSNR (dB)	Dtime (%)	BDBR (%)	BDPSNR (dB)	Dtime (%)	BDBR (%)	BDPSNR (dB)	Dtime (%)
TGM	1.60	-0.18	-39.0	0.46	-0.05	-24.9	1.81	-0.20	-50.0
M	0.59	-0.07	-37.3	0.19	-0.02	-21.0	0.47	-0.06	-42.4
A	0.12	-0.02	-25.9	0.08	-0.01	-17.8	0.16	-0.02	-35.2
CC	0.25	-0.02	-38.3	0.06	-0.01	-24.0	0.27	-0.02	-49.6

### 4.1 Individual approaches performance

Table 9 shows the individual results of the proposed approaches compared to the HEVC-SSC encoders, e.g. early CU depth level determination (ECUDLD) and adaptive intra mode selection (ADIMD). For ECUDLD approach in Table 9, 37.6% coding time has been saved on average. For most SC sequences, the ECUDLD approach reduces computational complexity by 25.9%–54.2%. These coding time reductions are particularly high for text and graphics sequences as “sc_SlideShow” (-54.2%), but they are still evident for animation sequences as “sc_robot” (-25.9%). The runtime reduction of “sc_SlideShow” and “Kimono1” sequences are larger than other test videos, because these sequences contain more homogeneous texture than other test videos. The runtime reduction is particularly high because a number of unnecessary depth levels are reasonably skipped in homogeneous region. Meanwhile, the PSNR loss is 0.12 dB, which is negligible. The above result indicates that the ECUDLD can efficiently skip unnecessary depth level of the HEVC-SSC. As for the ADIMD approach, about 24.0% coding time has been saved on average with the minimum of 17.8% in “sc_robot” and the maximum of 31.7% in “sc_SlideShow”. On the contrary, the average loss of PSNR is 0.04%, which is negligible. Therefore, the ADIMD can greatly save the encoding time while keeping the similar coding efficiency of HEVC-SSC.

### 4.2 Results of the overall method

In the following, we further show the coding performance of the proposed overall method in Table 10, which involves ECUDLD and ADIMD. It can be found that the proposed overall method saves 48.5% runtime on average, with the maximum of 66.3% for “sc_SlideShow” (TGM,1280×720) and the minimum of 35.2% for “sc_robot” (A,1280×720). The “sc_SlideShow” time reduction is larger than others, because “sc_SlideShow” contains more homogeneous texture than others. The computation reduction is high because unnecessary intra modes and depth level are skipped in the larger homogeneous region. Meanwhile, the average BDPSNR drop is 0.14 dB, which is negligible. Additionally, the results of proposed method for four categories of test sequences (“text and graphics with motion (TGM)”, “mixed content (M)”, “animation (A)”, and “camera-captured content (CC)”) are shown in Table 11. Table 11 gives the coding results of the two sub fast approaches and overall method. It can be seen that the coding runtime saving for the ECUDLD, ADIMD, and overall algorithm are 39.0%, 24.9%, and 50.0% for “TMG” sequences, 37.3%, 21.0%, and 42.4% for “M” sequences, 25.9%, 17.8%, and 35.2% for “A” sequences, 38.3%, 24.0%, and 49.6% for “CC” sequences, respectively. Those coding runtime reductions are particularly high for “CC” and “TGM” sequences, but they are still evident for “A” sequences. The above result analysis indicate that the proposed sub fast approaches and overall method can achieve a consistent gain in coding speed for different four categories sequences. From those results, it can be also observed that the proposed overall method primarily saves the encoding time with only about 0.16–1.81% RD performance loss for all the sequences. Therefore, the proposed overall method can significantly save the encoding time with similar RD performance.

**Fig 2:**
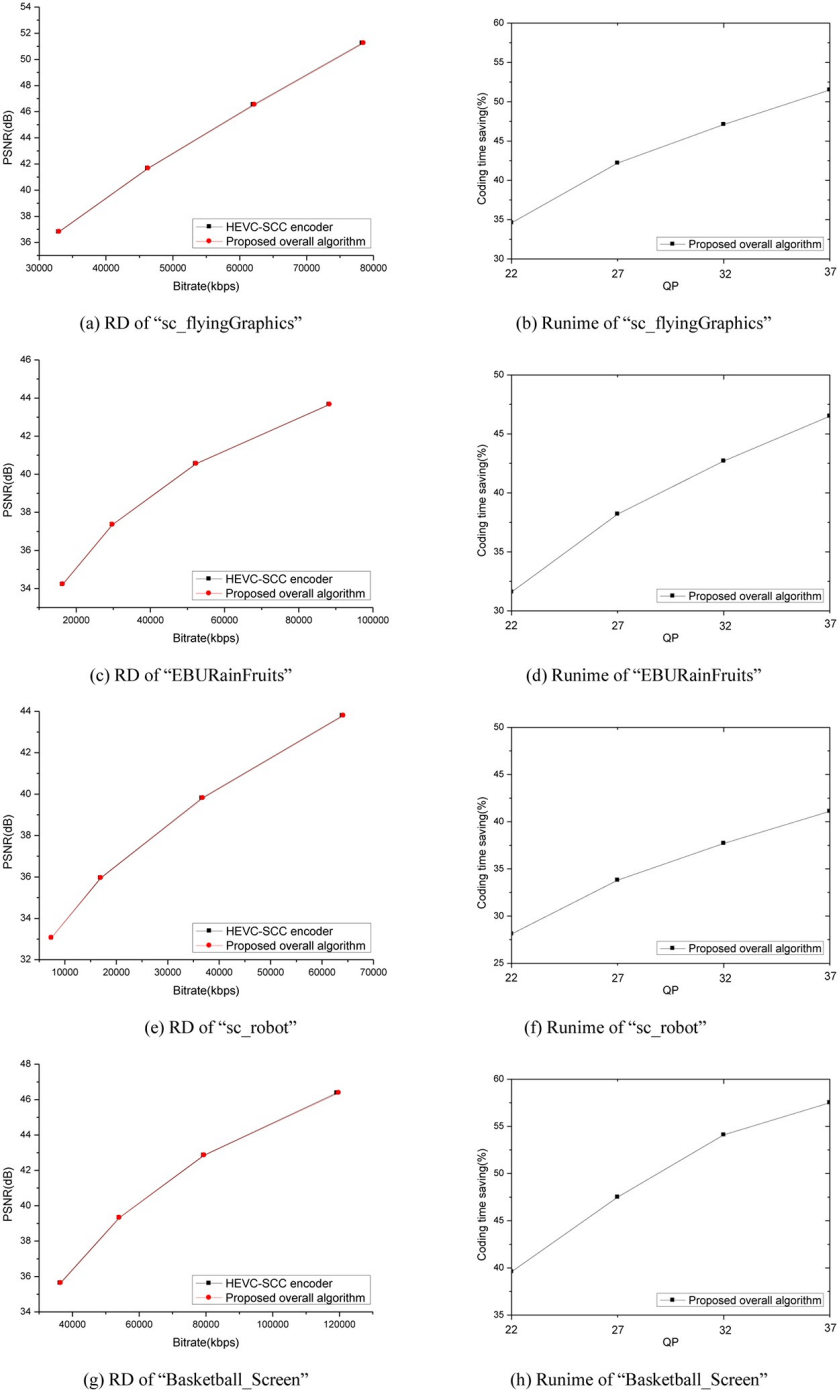
Experimental results of “sc_flyingGraphics” (TGM,1092×1080), “EBURainFruits”(CC,1092×1080),“sc_robot”(A,1280×720) and “Basketball_Screen” (M,2560×1440).

Fig 2 shows more detail simulation results of the proposed overall method for four typical test sequences “sc_flyingGraphics” (TGM,1092×1080), “EBURainFruits” (CC,1092×1080), “sc_robot”(A,1280×720), and “Basketball_Screen” (M,2560×1440). As shown in Fig 2, we can observe that the proposed overall method can achieve consistent runtime saving from low to high bitrate range with similar RD performance compared with HEVC-SCC. Moreover, with the decrement of compression bitrate and increment of QP value, the coding time savings increase in the curves. This is because that with the increment of QP values, the probability of only checking two depth levels (0,1) for homogenous treeblocks using ECUDLD, and the probability of testing Planar/DC Modes for screen content treeblocks using ADIMD are increased.

### 4.3. Performance comparison with the state-of-the-art methods

**Fig 3:**
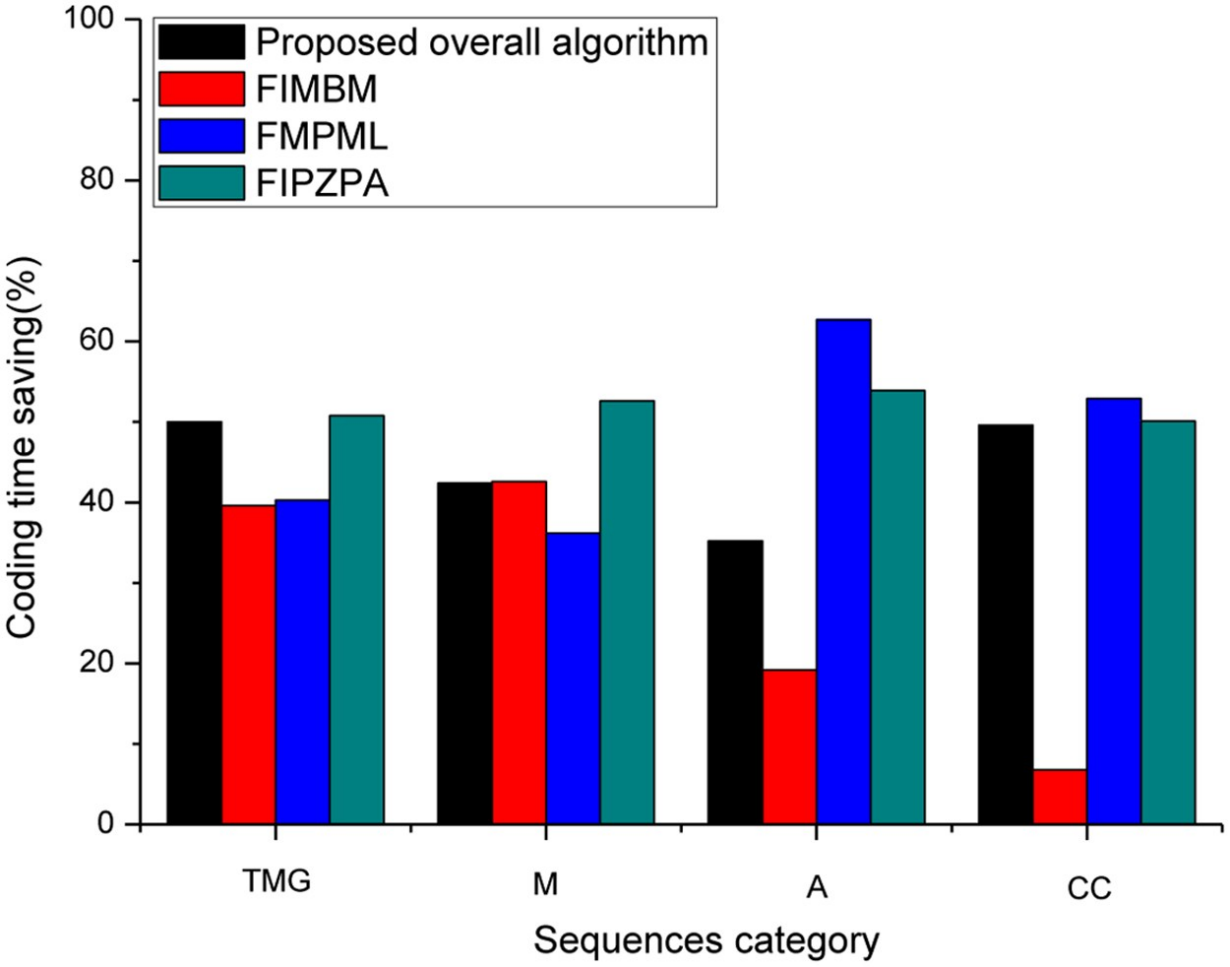
Coding time saving of proposed overall method, FIMBM, FMPML, and FIPZPA.

**Fig 4:**
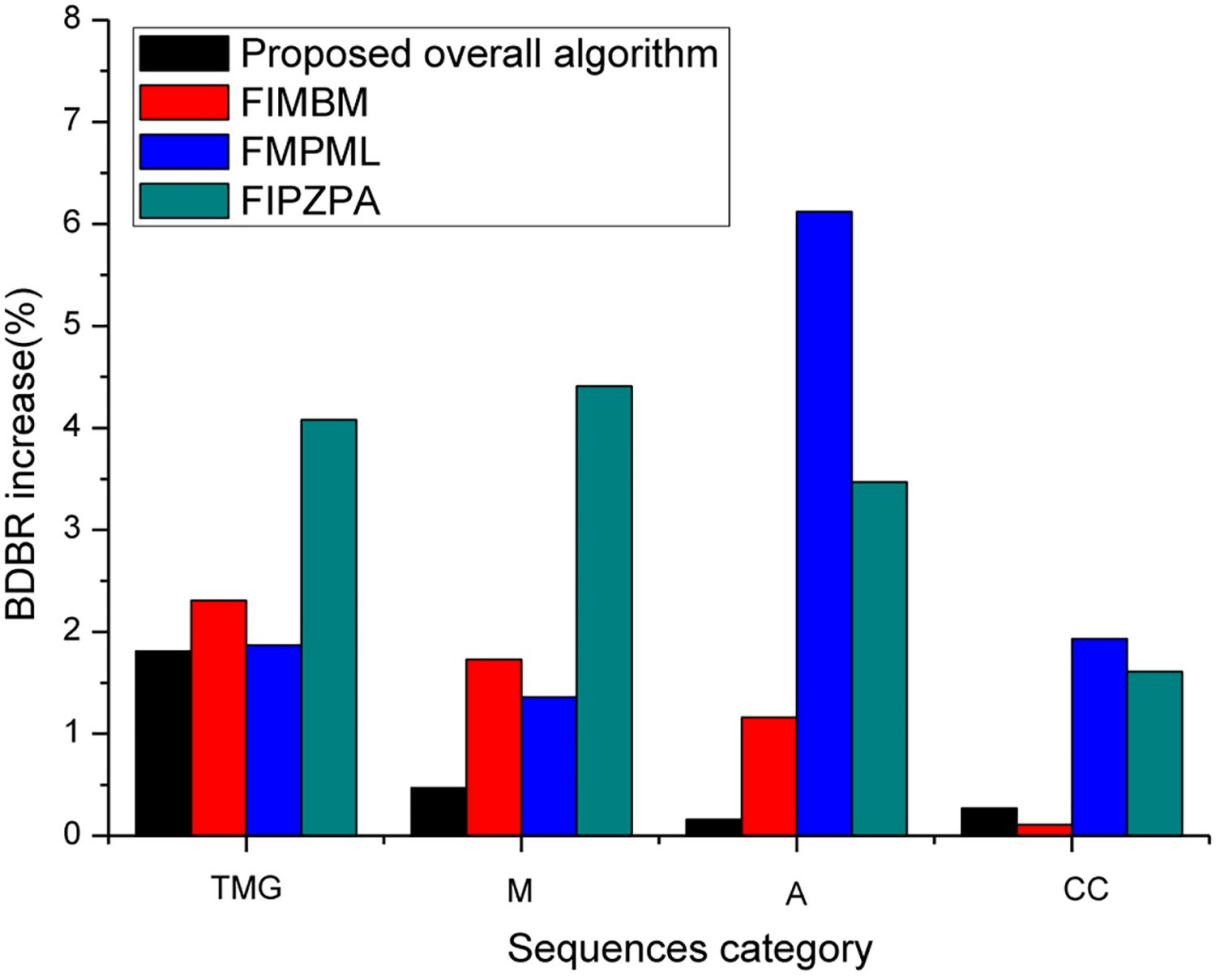
RD performance of proposed overall method, FIMBM, FMPML, and FIPZPA.

In addition to HEVC-SCC encoders, we compared the proposed overall method with recent state-of-the-art fast schemes with the SCM 5.2 implementation in Figs 3 and 4. These are FIMBM [25], FMPML [27], and FIPZPA [28], which are well-known fast and efficient methods for HEVC-SCC encoders. Among these four schemes, the proposed overall method has the better gain in RD performance compared with FIMBM, FMPML and FIPZPA. For four categories (TGM, M, A, and CC) of SC sequences, the proposed overall method achieves 35.2%-50.0% computation reduction on average with increasing only 0.2%-1.8% BDBR. As shown in Figs 3 and 4 the FIPZPA method performs the largest computation reduction, but its RD performance is poor. Compared to FIPZPA, the proposed overall method can achieve better RD performance. About 1.4%-3.9% BDBR can be further reduced in HEVC-SCC encoders. Meanwhile, the average computation reduction loss is negligible, 2.5% encoding time increase. Moreover, the proposed overall method can achieve the better gain (4.0%-10.0%) in coding time compared with FIMBM and FMPML schemes, and with a better coding efficiency (0.8%-1.3% BDBR decrease). The above simulation results indicate that the proposed overall method is efficient for all categories (TGM, M, A, and CC) of SC sequences and outperforms the recent state-of-the-art methods for HEVC-SCC.

## 5. Conclusion

In this paper, a fast intra mode decision method is proposed to reduce the complexity of SC compression, which comprises two approaches, i.e., early CU depth determination and adaptive intra mode selection. It makes use of the texture complexity classification of the SC treeblock to predict the current CU and early skip unnecessary intra mode. The experimental data shows that the proposed method can save about 48.5% coding time of the HEVC-SCC while keeping nearly the same RD performance.

## Supporting information

S1 File Encoder settings and get test videos. (ZIP)
