# Supplementary material for: Efficient intra mode decision for low complexity HEVC screen content compression
Source: PLoS One. 2019 Dec 31;14(12):e0226900. doi: 10.1371/journal.pone.0226900 (PMC6938359; doi:10.1371/journal.pone.0226900)
Supplement: S1 File — (ZIP) [file pone.0226900.s001.zip › S1File/Encoder settings.doc]

## Encoder settings for anchors

The following SCC specific encoder settings were used in generating the anchors:

- IntraBlockCopyEnabled = 1
- HashBasedIntraBlockCopySearchEnabled = 1
- IntraBlockCopySearchWidthInCTUs = -1
- IntraBlockCopyNonHashSearchWidthInCTUs = 1
- ColorTransform = 1
- PaletteMode = 1
- IntraBoundaryFilterDisabled = 1
- UseAdaptiveMvResolution = 1 (RA and LB)
- SelectiveRDOQ = 1

## In addition, lossy coding anchors with constrained IBC search area are also generated using the following IBC related encoder settings for 4:4:4 sequences:

- HashBasedIntraBlockCopySearchEnabled = 1
- IntraBlockCopySearchWidthInCTUs = 3
- IntraBlockCopyNonHashSearchWidthInCTUs = 1
