# Supplementary material for: Efficient intra mode decision for low complexity HEVC screen content compression
Source: PLoS One. 2019 Dec 31;14(12):e0226900. doi: 10.1371/journal.pone.0226900 (PMC6938359; doi:10.1371/journal.pone.0226900)
Supplement: S1 File — (ZIP) [file pone.0226900.s001.zip › S1File/Get test videos.doc]

## Test sequences

Test sequences are available from the following locations (please contact JCT-VC chairs for login information):

<ftp://hevc@ftp.tnt.uni-hannover.de/testsequences>

<ftp://hevc@ftp.tnt.uni-hannover.de/testsequences/FrExt-candidate-sequences/upload/screen_content/ScExt-TestSequences>

Note that test sequences from the EBU (i.e. with the string ‘EBU’ included in the test sequence name) are password protected. The password can be obtained by registering at the following location:

<http://tech.ebu.ch/testsequences/uhd-1_public_form>

The test sequences that are part of these common conditions are listed in Table 1 and Table 2.

**Table 1 - 4:4:4 Test Sequences**

| **Resolution** | **Sequence name** | **Category** | **fps** | **Frames to be encoded** |
| --- | --- | --- | --- | --- |
| 1920x1080 | sc_flyingGraphics_1920x1080_60_8bit*  sc_desktop_1920x1080_60_8bit  sc_console_1920x1080_60_8bit  ChineseEditing_1920x1080_60_8bit  MissionControlClip3_1920x1080_60p_8b444  EBURainFruits_1920x1080_50_10bit**  Kimono1_1920x1080_24_10bit*** | TGM  TGM  TGM  TGM  M  CC  CC | 60  60  60  60  60  50  24 | 0-299^*^  0-599  0-599  0-599  0-599  0-249**  0-119*** |
| 1280x720 | sc_web_browsing_1280x720_30_8bit  sc_map_1280x720_60_8bit  sc_programming_1280x720_60_8bit  sc_SlideShow_1280x720_20_8bit  sc_robot_1280x720_30_8bit | TGM  TGM  TGM  TGM  A | 30  60  60  20  30 | 0-299  0-599  0-599  0-499  0-299 |
| 2560x1440 | Basketball_Screen_2560x1440_60p_8b444  MissionControlClip2_2560x1440_60p_8444 | M  M | 60  60 | 322-621  120-419 |
| *Note that only the first 300 frames of this sequence are used.  **Note that only the first 250 frames of this 10-bit sequence are used, and InternalBitDepth is set to 8.  ***Note that only the first 120 frames of this 10-bit sequence are used, and InternalBitDepth is set to 8  TGM: Text and graphics with motion; M: mixed content; A: animation; CC: camera-captured content | | | | |

**Table 2** - 4:2:0 Test Sequences

| Resolution | Sequence name | Category | fps | Frames to be encoded |
| --- | --- | --- | --- | --- |
| 1920x1080 | sc_flyingGraphics_1920x1080_60_8bit_420*  sc_desktop_1920x1080_60_8bit_420  sc_console_1920x1080_60_8bit_420  ChineseEditing_1920x1080_60_8bit_420  MissionControlClip3_1920x1080_60p_8b420 | 4:2:0 TGM  4:2:0 TGM  4:2:0 TGM  4:2:0 TGM  4:2:0 M | 60  60  60  60  60 | 0-299*  0-599  0-599  0-599  0-599 |
| 1280x720 | sc_web_browsing_1280x720_30_8bit_420_r1  sc_map_1280x720_60_8bit_420  sc_programming_1280x720_60_8bit_420  SlideShow_1280x720_20  sc_robot_1280x720_30_8bit_420 | 4:2:0 TGM  4:2:0 TGM  4:2: 0TGM  4:2:0 TGM  4:2:0 A | 30  60  60  20  30 | 0-299  0-599  0-599  0-499  0-299 |
| 2560x1440 | Basketball_Screen_2560x1440_60p_8b420  MissionControlClip2_2560x1440_60p_8420 | 4:2:0 M  4:2:0 M | 60  60 | 322-621  120-419 |
| 1024x768 | ChinaSpeed_1024x768_30 | 4:2:0 A | 30 | 0-499 |
| *Note that only the first 300 frames of this sequence are used.  TGM: Text and graphics with motion; M: mixed content; A: animation; CC: camera-captured content | | | | |

Annex A includes md5sums for the test sequences in the common test conditions.

The following parameters are used to configure the encoder for a given test sequence:

- InputFile: Specifies the file name and path of a given test sequence.
- FrameRate: Specifies the frame rate of a given test sequence.
- SourceWidth: Specifies the width of a given test sequence.
- SourceHeight: Specifies the height of a given test sequence.
- FramesToBeEncoded: Specifies the encoded frame count for a given sequence.
- InputBitDepth: Specifies the bit-depth of a given test sequence.
- InternalBitDepth: Specifies the internal bit-depth of operation for the encoder for a given test sequence.

## Annex A. Test sequence md5sums

For each sequence included in the common test conditions, the ‘md5sum’ value is provided in Table 3.

**Table 3** - Test Sequences with md5sum

| format | file name | MD5 |
| --- | --- | --- |
| 4:4:4 RGB | sc_flyingGraphics_1920x1080_60_8bit_rgb.zip | 5ec2977e16da42a2d4373b893d7bd636 |
|  | sc_desktop_1920x1080_60_8bit_rgb.zip | 45d2d0ad013a5cb1313c5b542c98ec5f |
|  | sc_console_1920x1080_60_8bit_rgb.zip | 6181644352dc7194146fca0c4daf54ee |
|  | ChineseEditing_1920x1080_60_8bit_rgb.zip | d5100ab1dbbad87aba92364254eee82f |
|  | MissionControlClip3_1920x1080_60p_8b444.zip | 81dbb94053a737a203236c888808a9ae |
|  | EBURainFruits_1920x1080_50_10bit_444_rgb_ebu.zip | 6086e46bc8337b1f6752c51e25b9d0f7 |
|  | Kimono1_1920x1080_24_10bit_444_rgb.zip | 682e252e549a64d2e3f4676e3bf9ffab |
|  | sc_web_browsing_1280x720_30_8bit_300_rgb.zip | 04d5451b34bb601e4e83e3688643ecb3 |
|  | sc_map_1280x720_60_8bit_rgb.zip | fbb45233360302e115e2c331c16e534a |
|  | sc_programming_1280x720_60_8bit_rgb.zip | 8cef6e8149bef2903dc1f6cb4c7c46b0 |
|  | sc_SlideShow_1280x720_20_8bit_500.zip (RGB&YUV) | 2c73107851bc237be07d51297b526a0e |
|  | sc_robot_1280x720_30_8bit_300_rgb.zip | 36d3ef423954ee8d98deabb1b6a82477 |
|  | Basketball_Screen_2560x1440_60p_8b444.zip | 215784fd2cab1ade97dc740883c24c93 |
|  | MissionControlClip2_2560x1440_60p_8b444.zip | f535830c4ed233a9e998426c3b593294 |
| 4:4:4 Y’CbCr | sc_flyingGraphics_1920x1080_60_8bit_yuv.zip | d1ef8f76e0d0e025401b3cc03bde8986 |
|  | sc_desktop_1920x1080_60_8bit_yuv.zip | cf94582dc37dc62f8c0b3ac36894c8b5 |
|  | sc_console_1920x1080_60_8bit_yuv.zip | ad9fee7d7e32c891cf183b7ea43b325a |
|  | ChineseEditing_1920x1080_60_8bit.zip | 750dacd3c851765ba4e7f80a11459fca |
|  | MissionControlClip3_1920x1080_60p_8b444YUV.zip | 4b441b49f8c7fb7ae4031b266f330671 |
|  | EBURainFruits_1920x1080_50_10bit_444_ebu.zip | 7a73333b9e63938b5b194fc86718d30a |
|  | Kimono1_1920x1080_24_10bit_444.zip | 29490eccf2742c6e9fcaeb3165de4847 |
|  | sc_web_browsing_1280x720_30_8bit_300_444_r1.zip | 6d88218a4585f3e88e1c01cfcc3026ac |
|  | sc_map_1280x720_60_8bit_444.zip | 5bf31c2c90ca5e8baa30d6e90372b5e7 |
|  | sc_programming_1280x720_60_8bit_444.zip | cbea3b976a60aa69acbd48eb6a7ffdf7 |
|  | sc_SlideShow_1280x720_20_8bit_500.zip (RGB&YUV) | 2c73107851bc237be07d51297b526a0e |
|  | sc_robot_1280x720_30_8bit_300_444.zip | 0a03b4c5e508a4a7c00bd8331d926922 |
|  | Basketball_Screen_2560x1440_60p_8b444YUV.zip | 367bc916228f52a4c4ab66d19f0c406b |
|  | MissionControlClip2_2560x1440_60p_8b444YUV.zip | 3d71ecafc94a9b919eac3f0ef1270dae |
| 4:2:0 Y’CbCr | SlideShow_1280x720_20.zip | 34fbaf67d600a5d8bf06b3064934bb88 |
|  | ChinaSpeed_1024x768_30.zip | 2ce970e611113b249f0365cfb6e2ca5c |
|  | sc_flyingGraphics_1920x1080_60_8bit_420.zip | 8296308afe0421796a901d6f191f8a2a |
|  | sc_desktop_1920x1080_60_8bit_420.zip | 9d2de5da7982fbc2fd2d42437b3d9b6f |
|  | sc_console_1920x1080_60_8bit_420.zip | f946f37a415cf15ef0d6773b2a6e39fd |
|  | ChineseEditing_1920x1080_60_8bit_420.zip | dfb5754be4eafe02de48f80e6896ed87 |
|  | sc_web_browsing_1280x720_30_8bit_300_420_r1.zip | d1f20953a1fe477ab7ff77d0d833b812 |
|  | sc_map_1280x720_60_8bit_420.zip | 8d4f15bd463d156ea9032dc4bb356fed |
|  | MissionControlClip2_2560x1440_60p_8b420.zip | 6a3f7ebdb2e66ac7914a327f428caca0 |
|  | MissionControlClip3_1920x1080_60p_8b420.zip | f3df660027bc1cb71a9336f98c066ddb |
|  | sc_robot_1280x720_30_8bit_300_420.zip | 4e822bc43b49a3c64809cc54dbd8065f |
|  | sc_programming_1280x720_60_8bit_420.zip | c81a7dff676e8edcd4f58fd7a82c4369 |
|  | Basketball_Screen_2560x1440_60p_8b420.zip | 11e047d398f42a627ca5158dad20516e |
